# Risk of Preterm Birth among Secundiparas with a Previous Cesarean due to a Failed Vacuum Delivery

**DOI:** 10.3390/jcm12237358

**Published:** 2023-11-28

**Authors:** Sarit Helman, Muhammad Mahajna, Zvi Ehrlich, Miri Ratner, Sorina Grisaru-Granovsky, Orna Reichman

**Affiliations:** Department of Obstetrics and Gynecology, Shaare Zedek Medical Center, Faculty of Medicine, Hebrew University of Jerusalem, Jerusalem 9103102, Israelmiri.ratner@gmail.com (M.R.); sorina@szmc.org.il (S.G.-G.); orna.reich@gmail.com (O.R.)

**Keywords:** second-stage cesarean section, operative vaginal delivery, preterm birth

## Abstract

Background: Studies have found an association between second-stage cesarean sections (SSCSs) and subsequent preterm birth (PTB). We aimed to evaluate if secundiparas with previous second-stage cesarean sections due to a failed vacuum delivery (SSCS-F-VD) are associated with PTB in the subsequent delivery compared with secundiparas with previous spontaneous vaginal birth (SVB) at term. A secondary aim was to compare this association with secundiparas with a previous SSCS at term. Methods: A historical, prospective, longitudinal cohort study was conducted in a large tertiary university hospital between 2006 and 2019. Matched mothers who experienced first and second births at the indexed hospital, excluding those with a previous miscarriage or multiple pregnancy in either the first or second birth were grouped based on the mode of delivery and gestational week of the first birth. Results: Parturients with term SVB and term SSCSs were less likely to experience PTB in the following delivery compared with those who underwent an SSCS-F-VD, with 496/14,551 (3.4%) versus 6/160 (3.8%) versus 5/61 (8.2%), respectively, at *p* < 0.001. A logistic regression model revealed that secundiparas with previous SSCS-F-VD had an association with PTB in the following delivery compared with term SVB, with an OR of 2.756 (1.097; 6.922, *p* = 0.031). Conclusion: Previous SSCS-F-VD is associated with PTB in the following delivery, offering valuable insights for pregnancy management and patient counseling.

## 1. Introduction

Assisted vacuum delivery is an efficient intervention for a successful vaginal birth in cases of a prolonged second stage, a non-reassuring fetal heart rate, or limited maternal expulsive efforts. Potential neonatal and maternal complications limit its performance to medically indicated cases with appropriate fetal head position and cervical conditions [1,2]. A failure rate of 5–10%, requiring an emergent cesarean section (CS), is consistent among studies [3,4].

Previous studies found an association between a second-stage cesarean sections (SSCSs) and preterm birth (PTB) in the subsequent delivery [5,6,7,8,9,10,11]. This association may be the result of injury to the integrity of the cervix when attempting to extract a low, impacted fetal head during the SSCS. One of two mechanisms may explain this relationship: (1) inadvertently performing the incision too low within the cervix or (2) extensions of the lower segment incision that damage the cervix. Both mechanisms can injure the cervix and result, theoretically, in future cervix incompetence that is associated with PTB [8,12,13].

Secundiparas (parity = 2) subsequent to a failed-vacuum delivery represent the extreme of SSCSs, given that the fetal head was impacted in the pelvis prior to the cesarean, and as such, we assume that surgical complications, including uterine incision extension and difficulty locating the low-segment transverse from the cervix occur more often, thus placing this group at increased risk for PTB compared with other indications of SSCSs.

The primary aim of this study was to evaluate if secundiparas with a previous second-stage cesarean section due to a failed vacuum delivery (SSCS-F-VD) are associated with PTB in the subsequent delivery compared with secundiparas with a previous SVB at term. The secondary aim was to compare this association with secundiparas with a previous SSCS at term.

## 2. Methods

The study was conducted in a single, large tertiary university medical center, Shaare Zedek Medical Center (SZMC), Jerusalem, which is one of the two largest obstetric centers in the country, between January 2006 and December 2019. The obstetric profile of the study population is characterized by approximately 14,500 annual deliveries, which is estimated to comprise 10% of the national deliveries. Parity characteristics have been relatively stable throughout the last two decades; there have been ~25% nulliparous, ~18% grand multiparous (parity ≥ 6), ~12% cesarean deliveries, and ~5% vacuum deliveries. Over 95% of deliveries are funded by the national public insurance, managed by midwives and residents, and supervised by senior obstetricians.

The current study was a historical, prospective, longitudinal cohort study. All matched mothers who experienced a spontaneous onset of labor at first birth (P1) were screened for inclusion and exclusion criteria. Those with a live birth at ≥24 weeks of gestation at P1 and a second birth (P2) at the indexed setting were included. Parturients with a multiple gestation at either first or second birth, women with previous miscarriages, women with a non-vertex SVB, and women with an unplanned CS prior to the second stage of labor were excluded. Gestational weeks were categorized as PTB (<37 weeks) or term (37–42 weeks). Modes of delivery were grouped into five categories: SVB, vacuum delivery, planned CS at term, SSCS, and SSCS-F-VD. Matched mothers were grouped into six categories according to three criteria at P1: gestational week at birth, mode of delivery, and stage of CS: (1) term SVB, (2) term vacuum delivery, (3) term planned CS, (4) term SSCS, (5) term SSCS-F-VD, (6) PTB with either mode of delivery. Induction of labor (IOL) was not a criterion for subgrouping women, as we did not separate between indicated and spontaneous PTB.

Based on the obstetric profile of the study population, we expected to have 19,300 matched mothers in the study, of which 792 grouped in the planned CS, SSCS, and SSCS-F-VD groups. This estimation was based on the following: 200,000 births at SZMC during the study period (14,500 × 14), of which ~25% were primiparas, i.e., 50,000. Based on data from our medical center, 33% of primiparas do not return for the second delivery, and they deliver elsewhere [14]. Thus, of the 50,000, we expected only 33,000 (66%) to return for second birth. Hence, 3960 (33,000 × 0.12) was the estimated number of matched mothers who delivered at SZMC for both first and second births and underwent a CS at P1. We further excluded the 60% of parturients who underwent a CS during the first stage of labor [1,5]. As such, the estimated number of primiparas undergoing a planned CS or SSCS was 3960 × 0.4 = 1584; however, when excluding parturients undergoing a CS due to multiple gestations, with previous miscarriages, and those with missing data (approximately 50%), we estimated that 792 primiparas undergoing a CS would be included in the current study.

Data were extracted from a computerized database and updated in real time by midwives and obstetricians attending the labor and delivery. Most of the variables (11/12 (92%)) in the medical record were fixed, mandatory fields required to be completed before transferring the parturient to the postpartum ward. Data retrieved for both P1 and P2 included maternal age, gestational age at delivery, onset of labor (spontaneous, induction, or planned CS), mode of delivery (SVB, vacuum delivery, or CS), length of second stage at P1 (to classify the groups into term SSCS or term SSCS-F-VD and exclude women with CSs prior reaching full dilatation), newborn birthweight (BW), Apgar score at 1 and 5 min, and admission ward of the newborn.

Secundiparas with a history of SSCS-F-VD in their previous delivery were identified by searching the electronic medical database for ‘failed vacuum delivery’, as coded by the International Classification of Diseases (660.7), at P1. Most parturients with a breech presentation in our facility undergo a trial of external cephalic version at 37–38 weeks of gestation, and those who fail undergo a planned CS. Breech vaginal birth is performed under strict criteria. Indications for the CSs were documented for parturients grouped in the planned CS group: breech presentation, placental previa, macrosomia, maternal request, and a non-trial of labor due to a non-reassuring fetal monitor or other reasons.

Data were validated by defining distributions and quantifying missing values. Obstetric characteristics, stratified by first and second delivery, were presented as proportion, median, or mean with interquartile range or standard deviation, depending on the variable characteristics: categorical, ordinal, or continuous, respectively. Statistical significance was defined by a two-sided *p*-value ≤ 0.05 using the Chi-square test or Fisher exact test for categorical variables. The Wilcoxon signed-rank test was employed for ordinal or continuous variables with non-Gaussian distribution, and the Student paired t-test was utilized for continuous variables with a normal distribution. A univariate analysis followed by a binomial logistic regression were utilized with the aim of determining the effect of gestational week and mode of delivery at P1 on the likelihood of PTB at the second birth. Included in the model were gestational age and mode of delivery (P1) and maternal age (P2). Secundiparas with a history of SSCS-F-VD were associated with PTB compared with parturients with a history of SVB at term. Analyses were performed using IBM SPSS^®^ Statistics (version 29).

The study protocol and amendments were approved by the Institutional Review Board of the Shaare Zedek Medical Center (IRB # 0100-19). As this was a historical study, a waiver of informed consent was obtained. The manuscript is presented according to the STROBE guidelines [15].

## 3. Results

**Figure 1:**
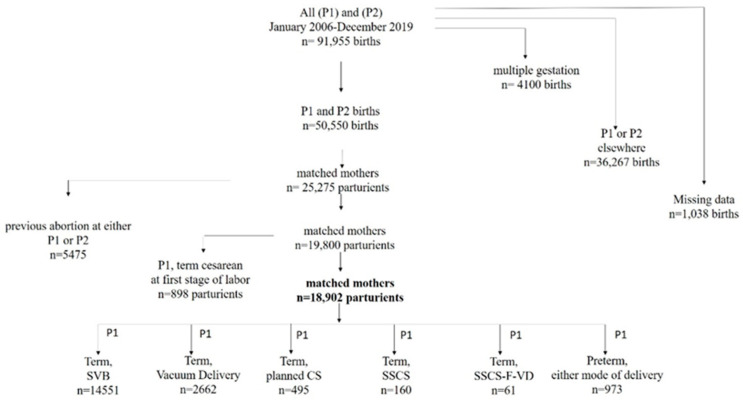
Flowchart of the study population. CS—cesarean section; P1—first birth, P2—second birth; SSCS—second-stage cesarean section; SSCS-F-VD—second-stage cesarean section due to a failed vacuum delivery; SVB—spontaneous vaginal birth.

During the 14 years of the study, a total of 91,955 primiparas and secundiparas gave birth at SZMC. After excluding multiple gestations of either primiparas or secundiparas, those who delivered elsewhere at first or second birth, and those with missing data regarding the inclusion and exclusion criteria, a total of 25,275 matched mothers who delivered both first and second births at SZMC remained, for a total of 50,550 births. Further exclusion of those with a previous abortion or who underwent a first-stage CS reduced the study group to 18,902 matched mothers. All six categories are presented in Figure 1, according to the three criteria at first birth: gestational week at birth, mode of delivery, and stage of CS. The vast majority of primiparas, or 14,551 (77%), culminated in a term SVB at the first birth compared with only a few parturients, or 61 (0.3%), who underwent an SSCS-F-VD.

Primiparas were an average of twenty-three years old and most had a second birth within two years of the first. PTB was more prevalent among primiparas, with 973 (5.1%), compared with secundiparas, with 822 (4.3%), *p* < 0.001. There was a low incidence of CSs in both the first (951, 5.0%) and the second (1024, 5.4%) birth, which is possibly explained by the exclusion of cases of term CSs in active labor prior to the second stage (Table 1). The prevalence of PTB at the second delivery was associated with the gestational week and mode of the previous delivery. Parturients with term SVBs and term SSCSs were less likely to experience PTB in the following delivery compared with those who underwent an SSCS-F-VD, with 496/14,551 (3.4%) versus 6/160 (3.8%) versus 5/61 (8.2%), respectively, *p* < 0.001. Women with a previous planned CS were significantly associated with PTB compared with secundiparas with a previous SVB at term, with an aOR of 1.925 (1.295; 2.860, *p* = 0.001). Secundiparas with a previous PTB had a prevalence of 20.4% for experiencing PTB in the second delivery (Table 2).

A binomial logistic regression was performed to determine the effect of the gestational week and mode of delivery at P1 on the likelihood of PTB at the second birth. Included in the model were gestational age and mode of delivery (P1) and maternal age (P2). The model included 18,900 cases; was statistically significant, χ2 (6) = 388.422, *p* < 0.001; explained 6.8% of PTBs at the second delivery; and correctly classified 95.7% of the cases. The calculated area under the ROC curve of the model was 0.653 (95% CI, 0.631 to 0.674), *p* < 0.001 (Table 3).

## 4. Discussion

We have shown in this study that secundiparas with a previous SSCS-F-VD are associated with PTB in the subsequent delivery compared with secundiparas with a previous SVB at term, with an OR of 2.756 (1.097; 6.922, *p* = 0.031). More importantly, this study highlights that secundiparas with a previous SSCS-F-VD have more than double the prevalence of PTB in the subsequent delivery compared with secundiparas with a previous SSCS for indications other than a failed vacuum delivery, at 5/61 (8.2%) versus 6/160 (3.8%), respectively. This finding suggests that women undergoing an SSCS-F-VD are a unique clinical subgroup among those with an SSCS, necessitating subgroup analysis. This is in contrast to previous studies that focused on the mode of delivery and PTB, categorizing women undergoing an SSCS as a homogenous group without clinical subgroupings [5,6,7,8,9,10,11]. The pathogenicity suggested for the association between SSCSs and PTB assumes that unintended lower extensions of the uterine incision are more prevalent among SSCSs compared with planned or first-stage CSs [12,16]. These extensions have the potential to damage the muscle body of the internal os and can, theoretically, cause impairment of cervical function in future pregnancies, resulting in cervical incompetence and PTB [17]. In addition, during SSCSs, it is often hard to discriminate between the lower uterine segment and the cervix; therefore, the uterine incision can potentially directly transect the cervix. These hypotheses are somewhat similar to the theory concerning the association between spontaneous PTB and cervical trauma due to cervical conization or manipulation during uterine evacuation [18]. Women subsequent to an SSCS-F-VD represent the extreme of SSCSs, given that the fetal head was impacted in the pelvis [19], and as shown by a recent study, surgical complications of SSCD-F-VDs are increased compared with the surgical complications of other causes of SSCSs [2]. 

A systematic review and meta-analysis aimed to study the association between the mode of delivery and PTB in the following delivery and identified 8 retrospective studies which included 10,079,942 women, reporting the aRR for a PTB in the following delivery. Five of the eight studies (62.5%) reported an increased risk of PTB subsequent to prior CDs, with an overall aRR of 1.12 (95% CI, 1.01–1.24, I2 = 99.1%) [20]. It is of note that the eight studies had a heterogenous population including multiparas, previous miscarriages, and previous PTBs, and there was no adjustment for confounders in all of the studies. Other studies focused on the associations of different stages of CSs and compared between planned CSs, first-stage CSs, and SSCSs and PTB in the following delivery [5,12]. Wood included 189,021 matched secundiparas for P1 and P2 in his cohort study, excluding pairs with PTBs at P1. Parturients were grouped as follows: (1) CS performed prior to labor (6346 (3.36%)), (2) first-stage CS (23,072, (12.21%)), (3) SSCS (8607 (4.55%)), (4) unknown stage of CS (6049 (3.2%)), (5) SVB (99,956 (52.88%)), and (6) operative vaginal delivery (44,991 (23.8%)). They found that parturients with an SSCS at the first delivery were associated with PTB prior to 32 weeks of gestation at the following delivery with an aOR of 2.44 (1.91; 3.10) [5]. Duration of the second stage was not found to be associated with PTB, and all other groups were not at risk for PTB. In their editorial, Berghella et al. commented that the limitation of the study was the fact that interval pregnancies, including possible late missed miscarriages, which are known risk factors for future PTB, were not reported or adjusted for [6]. In our study, we excluded women with previous miscarriages and, as such, were not confounded by this factor. We found an association, although not statistically significant, for PTB following an SSCS, with an aOR of 1.231 (0.541; 2.802, *p* = 0.620), which is possibly resultant of excluding the subset of parturients with an SSCS-F-VD from the group, as they have a high association with PTB. Another explanation could be that the association between SSCSs and PTB is correct, yet the sample size was insufficient to show significance. Interestingly, in our study, the group of planned term CSs was found to have an association with PTB compared with term SVB, with an aOR of 1.925 (1.295; 2.860, *p* = 0.001). As this finding was different from others [5], we verified that the main indications for the CSs in this group were appropriate for a planned term CS. The vast majority were breech presentation (312/482 (64.7%)), followed by macrosomia (60/482 (12.44%)), fetal distress (39/482 (8%)), and patient request (33/482 (6.8%)), and the remainder were individual cases of placental previa, previous myomectomy, previous pelvic trauma, and transverse lie. It is possible that this specific group is associated with PTB, not by a direct causation but as a mediator. For example, a malformed uterus is associated with both PTB and CSs. Therefore, the CS by itself is not the reason for the PTB, but rather, the malformed uterus is the reason for PTB.

Concurrent to the current study, a multicenter retrospective study, including women from the current study, focusing on a similar clinical question was published [21]. That study utilized different methodologies, as 17% of the study population had a previous vaginal birth and 30% had a prior miscarriage. In addition, only SSCSs and SSCS-F-VD were included. It showed that SSCS-F-VD was associated with PTB prior to 37 weeks of gestation with an adjusted odds ratio of 2.05 and a 95% confidence interval of 1.11–3.79, *p* = 0.02.

As PTB is known to be influenced by multiple factors, and given that known factors such as previous PTB, multiple pregnancies, and previous miscarriages were excluded or stratified in the current study, it is not surprising that a poor overall discriminatory ability of the binomial logistic regression model was identified, as seen by the calculated area under the curve (AUC) of the receiver operating characteristic (ROC) of 0.653 (95% CI, 0.631 to 0.674), *p* < 0.001, implying that there are other factors not included in the model that play a significant role in PTB that need to be uncovered in future studies. However, given that the aim of the study was to evaluate the contribution of each independent variable to the model and not only to discriminate between those who will experience PTB and those who will not, we believe that it is worthwhile to present the results of the model despite its poor discriminatory ability.

Future studies are needed to look in depth at surgical documentation of lower uterine extensions. This will enable analysis of the association of such extensions with preterm delivery in the following delivery and whether structural cervical damage can explain the association between SSCD and subsequent PTB.

The current study design excluded known risk factors for PTB, such as multiple pregnancy and previous miscarriage. The effect of parity was neutralized by including only matched primiparas and secundiparas. Each woman served as her own control, and as such, many of the confounding factors associated with PTB, such as socioeconomic status, BMI, genetic factors, chronic illness, smoking, and alcohol habits, that were not available or missing from the dataset were neutralized. The vast majority of variables analyzed (11/12) are mandatory fields in the electronic medical records and were therefore accessible. Based on the obstetric profile of the study population, we expected to have 19,300 matched mothers in the study, of which 792 grouped into the planned CS, SSCS, or SSCS-F-VD groups. In actuality, we had 18,902 matched mothers, 98% of the expected number, with 716 parturients who underwent a CS at first birth (90% of the estimated number). The similarity between the expected number of parturients to be included in the study and the actual number included strengthens the internal validity of the results, verifying that selection bias was not a significant issue.

The current study has several limitations. Factors that are associated with a PTB, such as a urinary tract infection, placental abruption or circumvallate placenta, polyhydramnios, previous cervical conization or LOOP excision, and systemic infections such as COVID or appendicitis, were missing and, therefore, not able to be adjusted for in the regression analysis. Furthermore, grouping IOL with any one of the six subgroups studied without analyzing it as an independent factor could have had a confounding effect on the association studied.

Single-center studies are homogenous by nature, unlike multicenter studies that have potential differences in obstetric management and treatment protocols, which could affect the external validity of the study. However, strict inclusion criteria of a narrowly defined group of parturients with similar characteristics that were described by others minimize the significance of this limitation. Further strengthening the external validity of this study was the strong association of PTB among secundiparas with a previous PTB that was similar to the documented literature. The disadvantage of a small sample size leads to the possibility of the study being underpowered to show a significant association.

## 5. Conclusions

The current study shows that secundiparas with a previous SSCS-F-VD are associated with PTB compared with secundiparas with a term SVB or an SSCS for an indication other than a failed vacuum delivery. This finding suggests that SSCS-F VD are a unique clinical subgroup among SSCSs. Thus, future studies should consider addressing this group as an independent subgroup of SSCSs, offering valuable insights for subsequent pregnancy management and patient counseling following an SSCS-F-VD.

## Figures and Tables

**Table 1:** Obstetric characteristics of 18,902 parturients for first and second delivery *.

		Parity2 N = 18,902	Parity1 N = 18,902	*p*-Value
Maternal characteristics/obstetrical history				
Maternal age at delivery, years, median (min, max)		25 (17, 51)	23 (15, 49)	<0.001
Gestational age at delivery, median (25th, 75th)		39 (21, 43)	40 (22, 43)	<0.001
Preterm delivery at <37 gestational weeks, (%)		822 (4.3%)	973 (5.1%)	<0.001
Onset of labor	Spontaneous N (%)	17,181 (90.9%)	15,981 (84.5%)	<0.001
	Induction N (%)	1094 (5.8%)	2316 (12.6%)	
	CS N (%)	627 (3.3%)	605 (3.2%)	
Mode of delivery	SVB	17,363 (91.9%)	15,225 (80.5%)	<0.001
	Vacuum delivery	514 (2.7%)	2724 (14.4%)	
	CS	1024 (5.4%)	951 (5.0%)	
Neonatal outcome				
Birthweight, median (25th, 75th)		3275 (3000, 3556)	3194 (2910, 3466)	<0.001
Macrosomia (>4000 g) N, (%)		415 (4.8%)	589 (3,1%)	<0.001
Female gender		9169 (48.5%)	9186 (48.6%)	0.435
Apgar at 5 min ≤ 7 N, (%)		367 (1.9%)	285 (1.5%)	<0.001
NICU N, (%)		410 (2.2%)	666 (3.5%)	<0.001

* Excluding parturients with a history of miscarriages, multiple gestations, or term CSs in active labor prior to SSCS. CS—cesarean section; NICU—neonatal intensive care unit; SSCS—second-stage cesarean section; SVB—spontaneous vaginal birth.

**Table 2:** Obstetric characteristics at first (P1) and second (P2) births of 18,902 parturients, grouped by gestational week and mode of delivery at first delivery (P1) *.

		P1 Term, SVB N = 14,551	P1 Term, Vacuum Delivery N = 2662	P1 Term, Planned CS N = 495	P1 Term, SSCS N = 160	P1 Term, SSCS-F-VD N = 61	P1 Preterm, Either Mode of Delivery N = 973	*p*-Value
P1 first birth								
P1 maternal age, years, median (min, max)		23 (15, 42)	24 (17, 44)	26 (17, 49)	25 (17, 42)	24 (18, 39)	23 (15, 41)	<0.001
P1 macrosomia (>4000 g) N, (%)		438 (3%)	80 (3%)	46 (9.3%)	20 (12.5%)	4 (6.8%)	1 (0.1%)	<0.001
P1 female gender		7296 (50%)	1122 (42.1%)	257 (51.9%)	54 (30.8%)	27 (44.3%)	430 (44.2%)	<0.001
P1 Apgar at 5 min ≤ 7 N, (%)		115 (0.8%)	46 (1.7%)	6 (1.2%)	2 (1.3%)	3 (4.9%)	113 (11.6%)	<0.001
P1 NICU N, (%)		93 (0.6%)	84 (3.2%)	14 (2.8%)	7 (4.4%)	12 (19.7%)	456 (46.9%)	<0.001
P2 second birth								
P2 onset of labor	Spontaneous	13,538 (93%)	2403 (90.3%)	270 (54.5%)	116 (72.5%)	45 (73.8%)	809 (83.1%)	<0.001
	Induction	805 (5.5%)	189 (7.1%)	32 (6.5%)	6 (3.8%)	3 (4.9%)	59 (6.1%)	
	CS	208 (1.4%)	70 (2.6%)	193 (39%)	38 (23.8%)	13 (21.3%)	105 (10.8%)	
P2 mode of delivery	SVB	13,980 (96.1%)	2358 (88.6%)	193 (39%)	64 (40%)	22 (36.1%)	746 (76.7%)	<0.001
	Vacuum delivery	200 (1.4%)	174 (6.5%)	42 (8.5%)	26 (16.3%)	12 (19.7%)	60 (6.2%)	
	CS	371 (2.5%)	130 (4.9%)	260 (52.5%)	70 (43.8%)	27 (44.3%)	166 (17.1%)	
P2 Preterm labor < 37 W		496 (3.4%)	89 (3.3%)	28 (5.7%)	6 (3.8%)	5 (8.2%)	198 (20.4%)	<0.001

* Excluding parturients with a history of miscarriages, multiple gestations, or term CSs in active labor prior to SSCS. CS—cesarean section; NICU—neonatal intensive care unit; SSCS—second-stage cesarean section; SSCS-F-VD—second-stage cesarean section due to a failed vacuum delivery; SVB—spontaneous vaginal birth.

**Table 3:** Risk determinants for preterm birth at second birth.

		Univariate OR (CI)	*p*-Value	* Multivariate aOR (CI)	*p*-Value
Factors of first birth (P1)					
Parturients grouped by time and mode of delivery	Term, SVB	Reference group			
	Term, vacuum delivery	0.980 (0.779; 1.233)	0.864	1.025 (0.814; 1.2991)	0.834
	Term, planned CS	1.699 (1.148; 2.514)	0.008	1.925 (1.295; 2.860)	0.001
	Term, SSCS	1.104 (0.486; 2.508)	0.813	1.231 (0.541; 2.802)	0.620
	Term, SSCS-F-VD	2.530 (1.009; 6.344)	0.048	2.756 (1.097; 6.922)	0.031
	Preterm, either mode of delivery	7.258 (6.063; 8.689)	<0.001	7.437 (6.208; 8.910)	<0.001
Factors of second birth					
Maternal age		0.970 (0.952; 0.989)	0.002	0.949 (0.930; 0.969)	<0.001

* Multivariate analysis combining maternal age at second delivery and grouping of parturients by gestational week and mode of delivery at P1. CS—cesarean section; SSCS—second-stage cesarean section; SSCS-F-VD—second-stage cesarean section due to a failed vacuum delivery; SVB—spontaneous vaginal birth.

## Data Availability

Data is unavailable due to privacy or ethical restrictions.
